# Experiments upon the Body of Morris, Executed Jan. 15, 1841, at Philadelphia, with the Post Mortem Examination

**Published:** 1841-01-23

**Authors:** 


*Experiments upon the body of Morris, executed Jan.* 15, 1841, *at Philadelphia, with the post mortem examination.*

[Reported for the Medical Examiner.]

For half a minute after the drop fell, the body remained motionless, then violent struggles with strong stertorous respiration and movements of right hand, erection of penis and escape of fluid: in a minute and a half, violent agitation of both feet; struggle ceased at the fourth minute; the inspirations became more laboured, and occurred at longer intervals, but still continued after the motions of the body had ceased. A deep inspiration was audible the fifth minute, the seventh, the eighth, and the ninth, when they also ceased.

The pulse during this period presented some remarkable changes: at a minute and a half it was at 30, firm, rising; at two minutes and a half 40, strong; at three minutes and a half 64, strong, somewhat irregular; at five minutes 64, still irregular; at five minutes and a half it suddenly rose to 120, not so strong; at seven minutes too frequent to be counted, much smaller; at eight minutes fell again to 72, irregular, moderately strong. Between eight and nine minutes it suddenly ceased, and at nine minutes could not be felt at either wrist.

At 5 minutes the heart was examined by the hand, reported to beat violently, 142 pulsations.

7 minutes, heart reported at 50.

8 minutes, heart ceasing to beat.

8½ minutes, ventricle beats feebly.

10 minutes, chest examined by auscultation, no sound either of heart or lungs.

At the end of 35 minutes the body was cut down, and immediately transported to the room in which preparations had been made for a series of galvanic experiments.

The apparatus for this purpose consisted of a battery belonging to Prof. Cresson, composed of 400 pairs of 3 inches square, and a battery of 350 pairs 7 inches by 3, belonging to Professor Hare. The former was so arranged that 200 or 400 pairs could be employed at will.

Immediately upon the removal of the dress, the chest was carefully examined by Drs. Gerhard and Ashmead; no respiratory murmur, no sound of heart.

At 40 minutes a vein in the left arm was opened by Dr. Klapp: a few drops of dark, venous blood were withdrawn with difficulty, and immediately coagulated.

An attempt was now made to force out through an elastic tube, a portion of the air contained in the chest, by pressure upon the thorax; about two cubic inches were collected in a vessel over mercury, which, upon analysis, was reported to contain less carbonic acid than is found in the air expired by a living individual. At forty-seven minutes the galvanic experiments conducted by Professors Mitchell and Cresson were commenced; a piece of sheet copper, of about four inches in width, was bent into a half collar, in order that, when fitted to the neck, flat surfaces would be in contact with the skin, over the two pneumogastric nerves—and a flat piece of the same metal was fitted to a handle to enable the operator to apply it at will to different regions of the abdomen, or to the extremities; both were so arranged that they could be readily adapted, by means of a small hand-vice, to either pole; the surfaces with which they were brought in contact were always previously moistened with a solution of muriate of ammonia.

One half the battery of Prof. Cresson was first employed, (200 pairs;) the positive pole was connected with the collar on the neck, which was permitted to remain stationary, while momentary applications, in quick succession, of the negative pole, were made to the hypogastric region; at each contact heaving of chest with a jerk; a lighted candle held to nostrils, very slightly affected. *Auscultation.*—Short, incomplete respiratory murmur during inspiration, none during expiration; no sound of heart.

At 50 min.—Poles reversed, (negative to neck;) action more marked; flame of candle puffed outwards, with slight noise during expiration; no effect during inspiration; signs on auscultation same as before; inspiratory murmur stronger, but still very incomplete. The collar connected with the negative pole was now transferred to forehead; the positive still applied intermittently to hypogastric region; twitching of muscles of face; slight opening of eye; no action of chest.

At 53 minutes the first experiment was repeated (positive pole to neck, half the battery) with similar effects, which were not, however so decided, as at 47 minutes.

54 minutes.—Whole battery: decided expiration and inspiration; upon auscultation, the inspiratory murmur was found more marked; no sound of heart.

56 minutes.—Another vein opened in arm; blood flowed more freely; about ℥ss.; coagulated in 12 minutes.

58 min.—Large battery of Professor Hare employed—(by some accident it had been deranged, and would not give a spark when two metallic poles were brought into contact)—negative pole to neck; positive pole to hypogastrium; no movement. An additional quantity of acid was added; still no movement. The positive pole was now transferred to thigh, slight contraction of muscles of this region—to foot, the leg was slightly elevated, the whole action was confined, in both instances, to the muscles of the lower extremity, those of trunk remaining unaffected.

1 h. 2 min.—Return to battery of Professor Cresson—whole power employed—negative pole to neck—respiration again induced, whether positive pole be applied to abdomen, thigh, or leg, but with diminished energy; very slight variation of the flame of a candle held close to nostrils.

1 h. 4 min.—Positive pole to neck; spark observed on applying negative pole to thigh; candle sensibly but feebly affected. *Auscultation;* slight inspiration heard by Dr. Ashmead.

1 h. 6 min.—Negative pole to neck; acid added to battery. Positive pole applied as before, at intervals, to hypogastrium. A candle being held to nostrils, flame first thrown *outwards*, then drawn *inwards* at each application; the expiration not only preceded the inspiration, but was more marked; on reversing the poles, *the expiration still preceded the inspiration*, and both were more decided, as tested by the candle.

1 h. 9 min.—An effort was made to collect a small quantity of the air expired, in a flaccid caoutchouc bag, which, on analysis, did not differ appreciably from atmospheric air.

1 h. 12 min.—Negative pole still to neck; the positive being applied to anterior surface of arm, flexes forearm, raises hand, and contracts fingers.

1 h. 13 min.—Action on arm feeble, with 400 pairs, mere twitching of muscles; action on chest barely appreciable; no respiration; on leg more marked, but still very slight, scarcely raising foot from table.

1 h. 16 min.—Skin dissected from left side of neck and inside of right thigh, flaps turned over, flat poles applied to subcutaneous cellular tissue, and covered by flaps; battery and poles as before; very slight muscular twitching in vicinity of poles; the poles were now removed, and again applied to the skin, the flaps being replaced in their natural position, and the muscular contractions were decidedly more marked.

1 h. 20 min.—Left pneumogastric* and right anterior crural nerves exposed, and surrounded by strips of tinfoil; poles as before; sparks on applying positive pole to tinfoil round anterior crural; no action, but if, without breaking the connexion, the hand of the operator be placed so as to make a communication between the positive pole and the skin of the anterior face of thigh, the current is felt to pass through

* The post mortem examination showed that the left superior laryngeal nerve had been taken up, and not the pneumogastric.

the hand, instead of being transmitted directly to the nerve.

1 h. 26 min.—Negative pole applied under right clavicle; positive pole to thigh, no effect—to leg, slight contraction of muscles of this region—over diaphragm, and by an incision to inferior surface of diaphragm, still no action.

1 h. 30 min.—The battery of Professor Hare was again tried, no deflagration where metallic poles brought into contact,—positive pole to neck; negative pole to leg; slight contraction of muscle of leg.

1. h. 35 min.—Body turned on belly; right sciatic nerve exposed; positive pole to left pneumogastric.* Negative pole to sciatic; (battery of Professor Cresson, 400 pairs)—mere twitching of muscles of back; where negative pole to integuments of thighs, action of flexor muscles more marked on left side.

The left sciatic was now similarly exposed, and connected with the negative pole, the positive still remaining attached to left pneumogastric; action more strong than when applied to opposite sciatic, but less than when applied to surface of skin over sciatic.

The right pnuemogastric was now exposed and connected with positive pole, while the negative was applied to sciatic of same side; twitching of contiguous muscles very feeble. Negative pole transferred to left thigh; effects more marked, but still slight; this side appears less exhausted. It may be well to mention, that when the negative pole was applied to right thigh, a slight motion was perceptible in the right hand.

1 h. 50 min.—Right phrenic nerve exposed and connected with positive pole; negative to thigh of same side; scarcely any movement—transferred to the left thigh, movement more marked.

A point was now adapted to positive pole, aud introduced into meatus of right ear—negative to corresponding side of mouth, over infra orbital nerve, and over supra orbital nerve, followed by slight twitching of muscles of these regions in each instance.

The point was introduced as near as possible to the trunk of seventh pair as it emerges—negative to tip of tongue, in contact however with lips; slight contraction of muscles of corresponding side of face, with possibly a tendency to protrusion of tongue, not sufficiently appreciable, however, to be relied upon.

*Post-mortem examination at* 8 *P. M.*, 6 *hours after execution, conducted by* Dr. McClintock, *in the presence of a number of Physicians, and a large Class of Students.*

*External appearances.*—Body strongly built; great rigidity of right arm, and of both lower extremities,—more marked on right side, on which the anterior crural nerve is exposed; left upper extremity perfectly flexible, presenting at the middle of the arm a marked depression produced by the cord with which it was bound; the biceps above this is flaccid—below, is hard and swelled; the welt is removed entirely by kneading the muscle, which now becomes uniformly soft; countenance natural; no injection of conjunctiva; no œdema; penis flaccid; some fluid, black blood, not coagulable, escaped from an incision which had been made to expose the spinal marrow below the occiput.

*Head.*—On removing scalp, great prominence of parietal insertion of temporal muscles, equally marked on both sides; bluish appearance beneath temporal fascia, as if from extravasation of blood; on dissecting up fascia, temporal muscles more highly coloured than usual, apparently swelled, offering no effusion either above or beneath them, but presenting an engorgement of their substance,—moist, and dotted with drops of blood on a transverse section.

Bones of cranium of an ivory hardness, but not inordinately thick for a negro. Dura mater moderately congested on both sides, but congestion more marked on left; a few glands of Pacchioni on external surface; superior longitudinal sinus contains a little fluid blood posteriorly; elsewhere empty, (subject on back;) numerous glands of Pacchioni.

*Brain, on removal of dura mater.*—Right hemisphere natural; the cortical substance of a few convolutions near the vertex of a marked rose colour; no sub-serous effusion: left hemisphere—pia mater more injected; moderate subarachnoid effusion.

Falx detached from before backwards; no blood in inferior longitudinal sinus; on separating hemispheres, superior surface of corpus callosum natural; perpendicular plane of left hemisphere more injected than right.

Section on level with superior surface of corpus callosum; general reddish appearance of cortical substance, and rose colour of medullary rather more marked on left side; septum lucidum slightly injected.

Lateral ventricles—right contains a small teaspoonful of limpid serum; left, a teaspoonful and a half coloured with blood, (collected and measured.) Plexus choroides, and velum interpositum of left side, injected to double the amount of those on right side.

No fluid in fifth ventricle; in third ventricle about thirty drops of limpid serum.

*Cornua.*—Hippocampus and plexus rather more injected on left side.

Pineal gland contained a little gritty matter.

Left thalmus and corpus striatum injected to double the amount of those of the right side, but still the injection is not very marked.

On removing brain, left base more vascular; no subserous effusion; about two teaspoonfuls of serum in occipital fossa.

*Cerebellum* rather below than above medium size in negro; vessels more injected on surface of left hemisphere; a greater degree of injection was also exhibited in the substance of left hemisphere.

* Superior laryngeal.

*Neck.*—No external appearance of cord; on dissecting muscles, they exhibited nothing to note except their deep colour, which pervades the whole muscular system; no ecchymosis; the arteries and veins of both sides were empty, and offered no alteration; the right phrenic nerve was found enclosed in tin foil; left superior laryngeal similarly so; a slight collection of viscid, frothy mucus in pharynx; œsophagus pale, healthy throughout; larynx natural; slight ossification of thyroid cartilage; trachea immediately below cricoid cartilage presents a rose coloured circle internally, of about eight lines in width; elsewhere, natural; spinal marrow at section between second and third cervical vertebræ appears unaltered; no fluid.

*Thorax.*—Lungs free from adhesions; no fluid in pleuræ; mottled colour; lobules separated by dark veins; posterior portion slightly darker than elsewhere, crepitant throughout.

The great vessels of the heart were tied, and this organ, together with the lungs, removed; no fluid in pericardium; on cutting through aorta, a considerable amount of black fluid blood escaped from its lower extremity; the right cavities appeared distended with blood.

On opening abdomen, smell of fresh meat; liver of a darker colour than usual, but not enlarged; no injection of peritoneal coat of intestines.

Examination resumed, Jan. 16, at 1 P. M., 23 hours after execution. Rigidity of body still exists, with exception of left arm.

*Heart.*—During the night the blood had escaped from the right cavities, which were flaccid; on incising aorta longitudinally, a large quantity of black fluid blood, containing bubbles of air, escaped; and on pressing ventricle, an additional amount of black, frothy blood, was forced out; the finger introduced into the incision penetrated readily into the ventricle, without encountering the semilunar valves; the incision being continued to display the valves, showed them unaltered in texture or colour; right ventricle contained a small quantity of similar blood; no coagulum in any of the cavities; with these exceptions, the heart, carefully examined, presented nothing to note.

*Liver.*—Natural size; dark, mahogany colour, firm; a piece, an inch square, being removed, and forcibly squeezed in the hand, only a few drops of black blood escaped.

Gall bladder contained a moderate quantity of mahogany coloured fluid bile; mucous membrane natural.

*Spleen.*—Natural size and colour, presenting a fissure, on inferior right side, one inch in length, and one-eighth to one-fourth of an inch in depth, such as might be made by a cut, lined with peritoneum between the two surfaces, of which cellular adhesions are found down to its bottom; substance more firm than usual.

Left kidney—darker colour than usual; consistence firm; difference of colour between two substances very slight.

Right kidney—normal size, offering anteriorly a lobulated projection; darker coloured than left; containing rather more blood.

*Stomach.*—Externally healthy; when opened, void of fluid, but coated with a thick, tenacious, frothy mucus, similar to that found in pharynx; rugosities exceedingly prominent; resembles tripe; edges of rugosities more coloured than other parts of mucous membrane, which gives, every where, strips of normal length.

*Small intestine.*—Duodenum covered with thick, tenacious fluid, resembling contents of gall bladder in colour, but more viscid; rest of small intestine presents nothing to note; glands of Bruner numerous and well marked; glands of Peyer not developed; the lower portion contains a dark, feculent matter.

*Large intestine.*—Caput coli distended with a dark greenish pultaceous mass; rest of colon contracted to one-fourth its natural size, and empty; bladder contains about two ounces of urine.

